# Efficacy of Bilberry and Grape Seed Extract Supplement Interventions to Improve Glucose and Cholesterol Metabolism and Blood Pressure in Different Populations—A Systematic Review of the Literature

**DOI:** 10.3390/nu13051692

**Published:** 2021-05-17

**Authors:** Teresa Grohmann, Caroline Litts, Graham Horgan, Xuguang Zhang, Nigel Hoggard, Wendy Russell, Baukje de Roos

**Affiliations:** 1 Rowett Institute, University of Aberdeen, Foresterhill, Aberdeen AB25 2ZD, UK; t.grohmann@abdn.ac.uk (T.G.); c.litts@rgu.ac.uk (C.L.); n.hoggard@abdn.ac.uk (N.H.); w.russell@abdn.ac.uk (W.R.); 2 Formerly Rowett Institute, University of Aberdeen, Aberdeen AB25 2ZD, UK; 3 Biomathematics & Statistics Scotland, Aberdeen AB25 2ZD, UK; g.horgan@abdn.ac.uk; 4 By-Health Ltd. Co, No.3 Kehui 3rd Street, No.99 Kexue Avenue Central, Luogang District, Guangzhou 510000, China; zhangxg2@by-health.com

**Keywords:** bilberry, grape seed, extracts, glucose, cholesterol, blood pressure, human intervention studies

## Abstract

Intervention with fruit extracts may lower glucose and lipid levels, as well as blood pressure. We reviewed the efficacy of bilberry and grape seed extracts to affect these outcomes across populations with varying health status, age and ethnicity, across intervention doses and durations, in 24 intervention studies with bilberry and blackcurrant (*n* = 4) and grape seed extract (*n* = 20). Bilberry and blackcurrant extract lowered average levels of glycated hemoglobin (HbA1c), at least in Chinese subjects, especially in those who were older, who were diagnosed with Type 2 Diabetes Mellitus (T2DM) and who were participating in longer-term studies. We also found good evidence that across studies and in subjects with hypercholesterolemia, T2DM or metabolic syndrome, intervention with bilberry and blackcurrant extract, and to some extent grape seed extract, significantly lowered total and low density lipoprotein (LDL) cholesterol levels after four weeks. Intervention with grape seed extract may reduce systolic and diastolic blood pressure in subjects with hypertension or metabolic syndrome. Differential responsiveness in cholesterol and blood pressure outcomes between stratified populations could not be explained by age, dose or study duration. In conclusion, bilberry and blackcurrant extract appears effective in lowering HbA1c and total and LDL cholesterol, whereas grape seed extract may lower total and LDL cholesterol, and blood pressure, in specific population groups.

## 1. Introduction

In 2014, 422 million people, nearly 8.5% of the world’s population, were diagnosed with Type 2 Diabetes Mellitus (T2DM). Within 20 years, this number is expected to double, increasing the burden on healthcare systems, partly because of increased co-morbidities such as cardiovascular diseases (CVD) [1].

Daily consumption of fruits and green leafy vegetables can reduce T2DM risk and improve metabolic health outcomes [2]. Increasing evidence also suggests that fruit extracts may help prevent the onset of diabetes, possibly due to their flavonoid content including flavan-3-ols and anthocyanins [3]. Anthocyanins complexed with sugar moieties, such as rutinoside, and polymeric flavan-3-ols, represent a major proportion of flavonoids in fruits [4,5]. Many of these compounds have been found to transit the small intestine intact, after which they can be metabolized by the colonic microbiota [6]. Whilst the bioavailability of intact dietary flavan-3-ols and anthocyanins is relatively low, microbiota-derived metabolites are absorbed more easily and circulate in plasma for significantly longer periods of time [5,6]. Therefore, it has been suggested that microbiota-derived metabolites may contribute to the anti-diabetic effects of fruit, vegetable or fruit extracts [5,6].

The mechanism by which dietary flavonoids affect cardiometabolic outcomes is dependent on the concentration of flavonoids in the diet, on genetic factors that determine enzyme activity, on gut microbiota composition and on lifestyle, which all are highly individualized factors [7]; such factors tend to be overlooked in population-based studies [8]. Therefore, a more in-depth analysis of study population characteristics is required to better understand the variation in responsiveness to dietary interventions with flavonoids.

Higher dietary intakes of anthocyanins and catechins have been associated with a significantly lower risk of T2DM in 60,586 women over a 20-year observation period [9]. Bilberries are one of the richest natural sources of anthocyanins [10,11], whereas grape seeds contain the highest levels of total procyanidins and procyanidin dimers in fruits and vegetables [12]. However, previous studies evaluating the anti-diabetic and cholesterol-lowering effects of bilberry and grape seed extracts in humans have been inconclusive, mainly due to a large variability in outcomes within and between study populations. Often, the heterogeneity in study outcomes was not explored. A meta-analysis by Zhu et al. [13] concluded that dietary interventions with cranberries or blueberries had no effect on cholesterol levels, but intervention with whortleberry and bilberry improved cholesterol levels in subjects at risk of cardiovascular disease, and in subjects with metabolic syndrome and hypercholesterolemia. A systematic review by Woerdeman et al. [14] concluded that interventions with grape polyphenols did not significantly affect glycemia, insulin sensitivity, cholesterol levels or blood pressure, arguably due to differences in study quality, study populations and study durations. A recent meta-analysis, including meta-regression and subgroup analysis to identify sources of heterogeneity in previous reviews, concluded that intervention with grape seed extract decreased LDL cholesterol and triglycerides across studies, and LDL and total cholesterol in studies with less than 10 weeks of intervention, and in those studies providing <300 mg/d of grape seed extract [15]. The meta-analysis by Zhang et al. [16] concluded that grape seed extracts decreased systolic and diastolic blood pressure, with effects being significantly more pronounced in subjects with metabolic syndrome and in pre-hypertensive individuals. Interestingly, intervention with grape seed extract did not affect blood pressure in hypertensive subjects, arguably due to variance in study designs and bias arising from a low sample size [16].

The aim of this critical and systematic review was to investigate the efficacy of, and responsiveness to, different doses and intervention regimes of bilberry extract and grape seed extract to improve glucose and cholesterol metabolism, and blood pressure, in different populations.

## 2. Materials and Methods

The databases Cochrane Library, Ovid Medline (Embase 1996–2021, and Ovid Medline^®^ without revisions 1996–Week 5 2021) and Scopus were consulted up until January 2021 by two independent researchers (T.G. and C.L.). The following search terms were explored within titles, keywords and abstracts: (grape adj1 seed AND extract AND vitis adj1 vinifera) OR (bilberry AND extract AND vaccinium adj1 myrtillus) AND (human AND human adj1 study AND obes* AND Type 2 Diabetes) AND (blood adj1 glucose AND glycated h*emoglobin AND HbA1c) OR (cholesterol) OR (systolic AND diastolic).

Studies fulfilling the following criteria were included in the review: availability of a full-text English publication of the study; studies with a long-term intervention (i.e., ≥4 weeks) with bilberry and/or grape seed extract or a combination of either extract with other fruit extracts; studies executed in healthy subjects or those diagnosed with T2DM, metabolic syndrome, hypercholesterolemia or mild hypertension as classified by the authors of respective studies; studies with a randomized, placebo controlled and single- or double-blind design; studies measuring the following outcomes: fasting blood glucose, HbA1c, total cholesterol, HDL and LDL cholesterol, and systolic and diastolic blood pressure. Studies using fresh or frozen fruits, fruit juices, as well as acute studies and those that had exclusively recruited smokers were excluded from this review.

The following data were extracted from the selected studies: first author name, study duration, type of extract, information on supplement formulation, intervention dose, study design (cross-over/parallel), blinding, randomization, total number of subjects, number of subjects in the intervention and placebo group, number of drop-outs, male/female ratio, age range and BMI range of subjects, health condition, the calculated average percentage change for each of the outcomes between the end of the study and baseline for the intervention and placebo groups, and reported *p*-values.

The quality of studies was evaluated using the Cochrane method for bias evaluation [17]. The PRISMA checklist was followed to ensure review quality. We calculated a Spearman correlation matrix to explore which factors, such as intervention, dose, study duration, BMI and age were correlated with specific study outcomes, such as fasting blood glucose, HbA1c, total cholesterol, LDL and HDL cholesterol, systolic and diastolic blood pressure. The statistical and visual analysis of data was performed using R, version 4.0.2, and R Studio version 1.2.5042 [18]. The R packages used were corrplot [19] and ggplot2 [20]. No post-hoc test, such as Bonferroni, was applied to correct for the number of correlations as this may have increased the risk of introducing Type 1 errors in an exploratory analysis. Instead, we decided to set the level of significance at *p* ≤ 0.01.

## 3. Results

### 3.1. Study Selection

**Figure 1:**
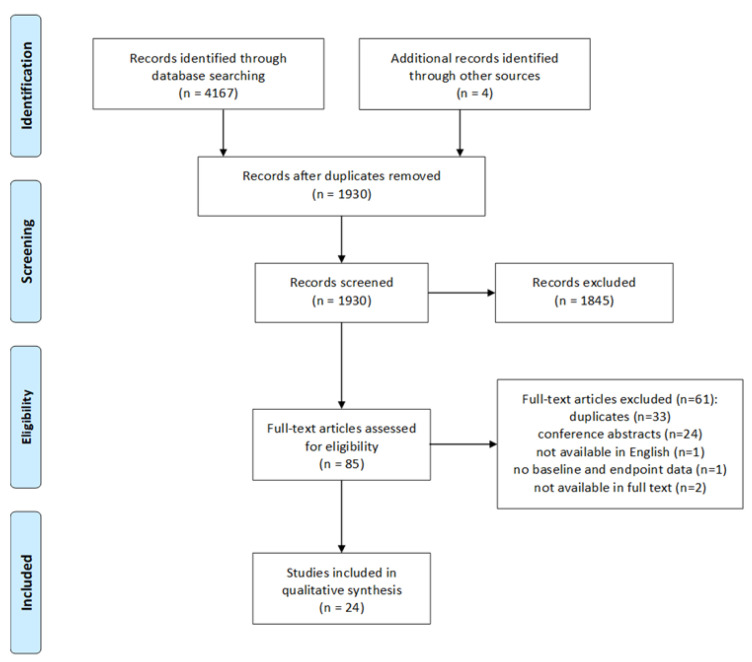
Process flow chart of the systematic literature search.

The results of the literature search and selection process are shown in Figure 1. In total, 4167 records were obtained from the search, and four additional records were identified through reference lists from other reviews. After initially removing duplicate records (*n* = 2241) based on titles and abstracts, a further 1845 records were excluded as these did not assess interventions with bilberry or grape seed extracts, or were studies which performed in vitro or in animal models. The remaining 85 records were further analyzed for full-text availability. We identified and removed 24 records, which were conference abstracts or registered clinical trials without publications, and removed 33 duplicate records. We also excluded one record as it was not available in English, one record as it did not provide baseline and endpoint data, and two records as these were not available in full text. In total 24 studies were analyzed for this review.

### 3.2. Study Characteristics

The main characteristics of the 24 selected studies are summarized in Table 1. The studies were performed in nine different countries. The number of volunteers per study ranged from 18 to 160. The study participants were either healthy (*n* = 3 studies), or healthy and in different stages of menopause (*n* = 2 studies), diagnosed with T2DM (*n* = 4 studies), diagnosed with metabolic syndrome (*n* = 5 studies), diagnosed with hypercholesterolemia (*n* = 5 studies), or diagnosed with mild hypertension (*n* = 5 studies). The BMI range across studies was 18 to 39 kg/m^2^, and the age range of study participants was between 13 and 75 years. Study populations in the USA had on average the highest BMI (age: 48 ± 4 years, BMI: 33 ± 3 kg/m^2^), whilst study populations in Japan had on average the lowest BMI (age: 52 ± 2 years, BMI: 23 ± 1 kg/m^2^). The youngest study population was recruited in Iran (age: 39 ± 15 years, BMI: 30 ± 4 kg/m^2^). Four studies recruited only women, while two studies did not identify the subjects’ sex. The majority of studies (*n* = 20) performed an intervention with grape seed extract, testing a supplement dose of 100 mg to 1500 mg per day. Four studies evaluated the effect of bilberry and blackcurrant extract at a dose of 320 mg per day. The duration of the intervention period across studies ranged from 4 to 52 weeks. Four studies employed a crossover design where intervention periods lasted four to eight weeks.

### 3.3. Evaluation of Study and Reporting Quality

**Figure 2:**
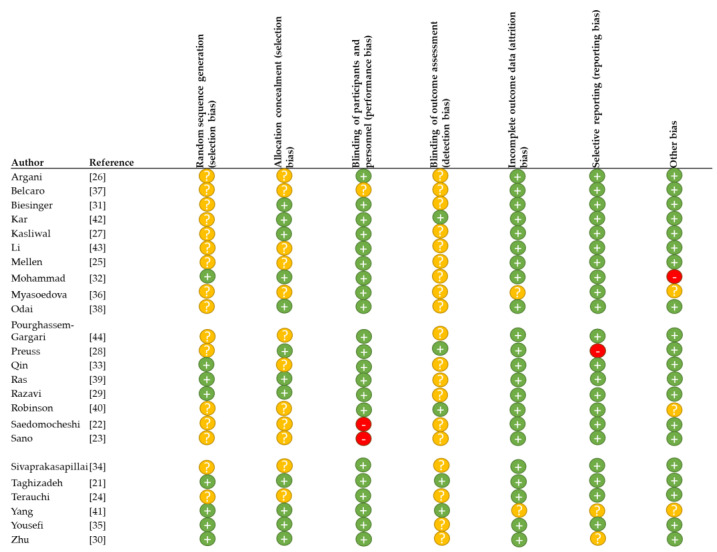
Cochrane evaluation of study bias; ”+” low risk of bias, “?” unclear risk of bias, “-“ high risk of bias.

The Cochrane bias evaluation was performed to evaluate study and reporting quality (Figure 2) [17]. The method for randomization was not described in 16 out of 24 studies, and concealment of intervention allocation was not described in half of the studies (*n* = 12). Participants and researchers were blind to the intervention sequence in most studies (*n* = 21). One study did not describe the blinding process [37], and two studies described issues which may have affected the blinding: an unequal number of capsules for the intervention and placebo [22] and blinding of researchers but not participants to the intervention sequence [23]. Two studies reported dropouts, but did not mention baseline population characteristics after dropouts [36,41]. One study only reported blood pressure outcomes for selective individuals with hypertension [28]. We detected gender imbalance between intervention and placebo groups in two studies [32,40]. In one study, baseline levels of insulin and HOMA-IR, the main outcomes in this study, were significantly different between intervention and placebo groups at baseline [32]. Two studies reported unequal participant numbers in intervention and placebo group after dropouts [36,41]. Half of the studies did not report withdrawals or dropouts; the reported drop-out rates ranged from 1% to 38% (Table 1).

### 3.4. Correlation Analysis

**Figure 3:**
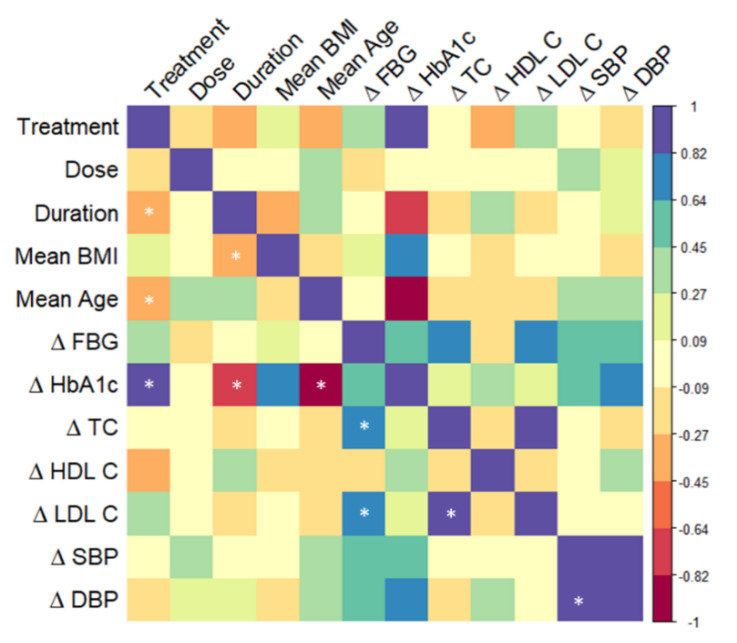
Correlation heatmap of study characteristics, subject characteristics and percentage change in fasting blood glucose, HbA1c, cholesterols and blood pressure levels across 24 studies. The treatments were expressed as numbers (bilberry and blackcurrant = 1, grape seed = 2). Mean BMI and age were calculated for each study population where reported. FBG—fasting blood glucose, HbA1c—glycated hemoglobin, TC—total cholesterol, HDL C—high density lipoprotein cholesterol, LDL C—low density lipoprotein cholesterol, SBP—systolic blood pressure, DBP—diastolic blood pressure. The matrix was calculated using corrplot [19] in R studio. * *p* < 0.01.

A correlation analysis of factors including study design, subject characteristics and study outcomes was performed across the 24 studies to assess whether any of these factors could explain the efficacy of the bilberry and grape seed interventions to affect glucose and cholesterol metabolism, and blood pressure, in stratified groups of volunteers (Figure 3).

When considering significant correlations (*p* ≤ 0.01) across studies, we observed that average changes in HbA1c levels were positively correlated with treatment (R = 0.84; *p* = 0.008), indicating that bilberry and blackcurrant extract (treatment 1) may be more effective than grape seed extract (treatment 2) in lowering HbA1c levels. In addition, average changes in HbA1c were negatively correlated with age (R = −0.88; *p* = 0.003), and with study duration (R = −0.69; *p* = 0.005), indicating that larger reductions in HbA1c levels were observed in subjects who were older and participating in longer-term studies. Average changes in levels of fasting blood glucose were positively correlated with average changes in total cholesterol (R = 0.78; *p* = 0.007) and LDL cholesterol levels (R = 0.7; *p* = 0.002), suggesting that modulation of these outcomes could share common mechanistic pathways, such as those involved in increasing insulin sensitivity. Average changes in total and LDL cholesterol were positively correlated (R = 0.87; *p* < 0.001), as were average changes in systolic and diastolic blood pressure (R = 0.88; *p* < 0.001), which is to be expected, as these outcomes are physiologically linked. Furthermore, treatment was negatively correlated with study duration (R = −0.45; *p* = 0.001) and age of subjects (R = −0.41; *p* = 0.003), indicating differences in study design and study populations between studies with either bilberry and blackcurrant extract or grape seed extract. Study duration was also negatively correlated with average BMI (R = −0.45; *p* = 0.002), indicating that participants with a lower BMI took part in longer-term interventions.

### 3.5. Efficacy of Grape Seed or Bilberry and Blackcurrant Extract Supplements to Modulate Glucoses Metabolism

**Figure 4:**
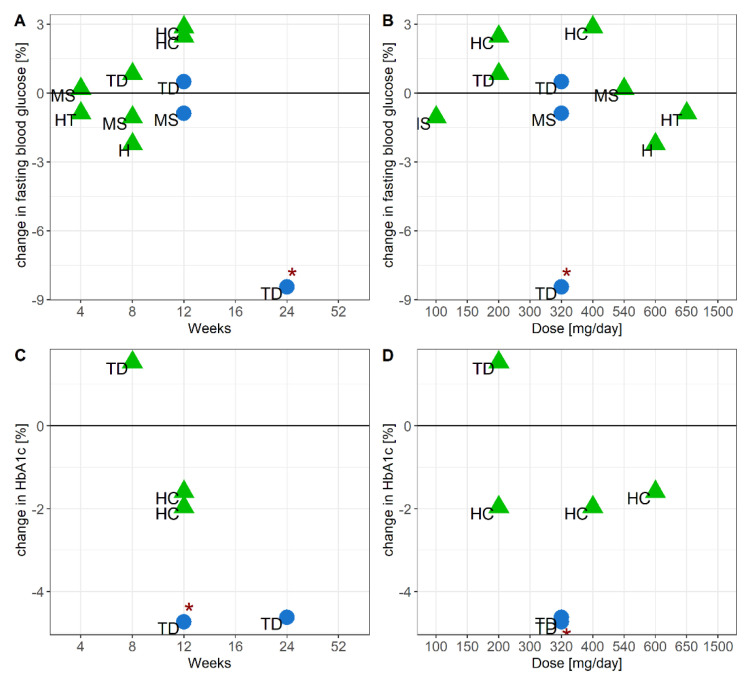
Relationship between the average change in fasting blood glucose and study duration (**A**), or dose (**B**), and between the average change in HbA1c levels and study duration (**C**), or dose (**D**). ● = bilberry and blackcurrant extract (blue), ▲ = grape seed extract (green), study population: H = healthy, HC = hypercholesterolemia, HT = hypertension, MS = metabolic syndrome, TD = Type 2 Diabetes Mellitus. * published *p*-value < 0.05.

Nine studies—six studies with grape seed extract [21,23,25,31,32,44] and three studies with bilberry and blackcurrant extract [33,41,43]—measured fasting blood glucose levels as an outcome. Five studies—three studies with grape seed extract [23,27,44] and two studies with bilberry and blackcurrant extract [41,43]—measured HbA1c as an outcome. None of the studies with grape seed extract observed significant changes in levels of fasting blood glucose or HbA1c, which may be linked to the fact that the majority of these studies were performed in healthy subjects (Figure 4). One out of three studies with bilberry and blackcurrant extract measuring fasting glucose as an outcome reported a significant reduction of 8.5% in levels of fasting blood glucose in subjects diagnosed with T2DM [43]. One out of two studies with bilberry and blackcurrant extract measuring HbA1c as an outcome reported a significant decrease of 4.7% in levels of HbA1c in subjects diagnosed with pre-diabetes and T2DM [41]. There was no evidence for a dose-response effect across studies with either grape seed or bilberry and blackcurrant extract (Figure 4).

### 3.6. Efficacy of Grape Seed or Bilberry and Blackcurrant Extract Supplements to Modulate Cholesterol Metabolism

**Figure 5:**
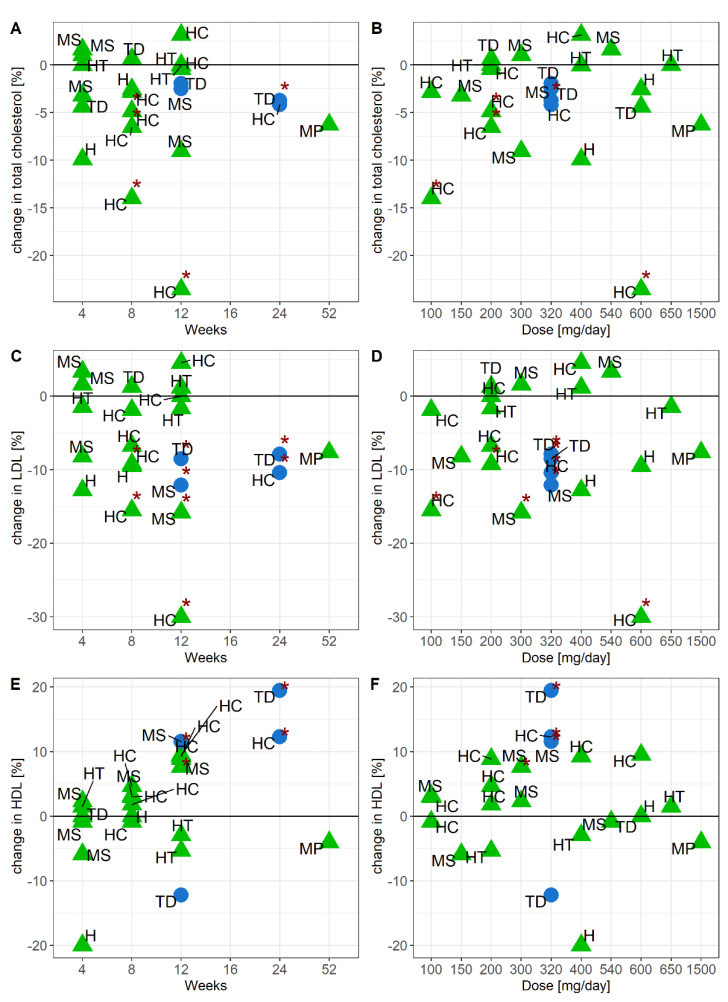
Relationship between total cholesterol and study duration (**A**), or dose (**B**), LDL cholesterol and study duration (**C**), or dose (**D**), HDL cholesterol and study duration (**E**), or dose (**F**). ● = bilberry and blackcurrant extract (blue), ▲ = grape seed extract (green), study population: H = healthy, HC = hypercholesterolemia, HT = hypertension, MP = mixed postmenopausal, MS = metabolic syndrome, TD = Type 2 Diabetes Mellitus. * published *p*-value < 0.05.

Twenty studies—sixteen studies with grape seed extract and four studies with bilberry and blackcurrant extract—measured effects on cholesterol outcomes. All four bilberry and blackcurrant extract studies [30,33,41,43] and five of the sixteen grape seed extract studies [26,27,28,29,35] observed significant improvements in at least one cholesterol marker. Significant reductions in total and LDL cholesterol were observed across studies, and in different study populations where subjects had been diagnosed with hypercholesterolemia, T2DM or metabolic syndrome, in those studies that lasted four weeks or longer. Longer study durations did not reduce total and LDL cholesterol further. The effect of intervention with either grape seed or bilberry and blackcurrant extract on HDL cholesterol was more variable. Only one study with grape seed extract [35], and three intervention studies with bilberry and blackcurrant extract [30,33,43], showed a significant average increase in HDL cholesterol, but the response was not linked to study design or population characteristics, or other study outcomes. Dose did not affect the response in cholesterol levels (Figure 5).

### 3.7. Efficacy of Grape Seed and Bilberry and Blackcurrant Extract Supplements to Modulate Blood Pressure

**Figure 6:**
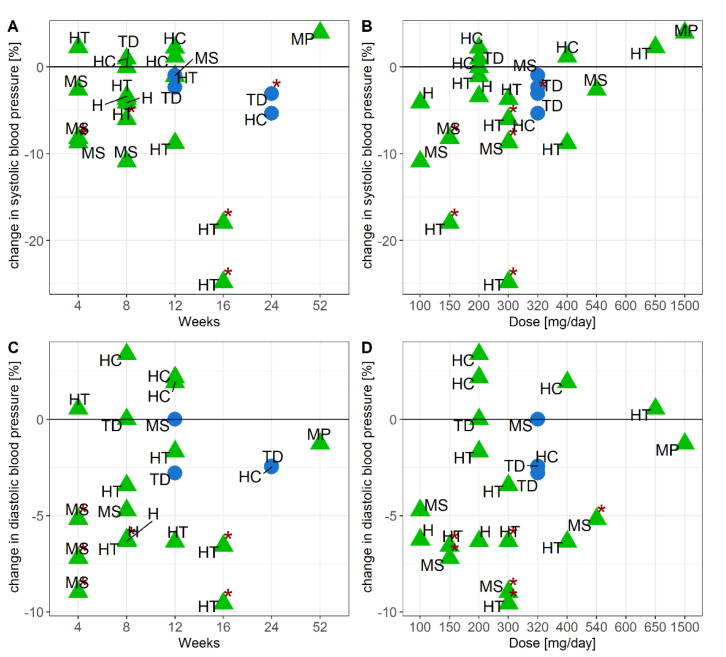
Relationship between systolic blood pressure and study duration (**A**), or dose (**B**), diastolic blood pressure and study duration (**C**) or dose (**D**). ● = bilberry and blackcurrant extract (blue), ▲ = grape seed extract (green), study population: H = healthy, HC = hypercholesterolemia, HT = hypertension, MP = mixed postmenopausal, MS = metabolic syndrome, TD = Type 2 Diabetes Mellitus. * published *p*-value < 0.05.

Systolic and diastolic blood pressure was monitored in 17 studies—thirteen with grape seed extract and four with bilberry and blackcurrant extract (Figure 6). Four of the thirteen studies with grape seed extract [31,34,37,40] reported a significant reduction in both systolic and diastolic blood pressure in subjects diagnosed with metabolic syndrome or hypertension, whereas only one study with bilberry and blackcurrant extract reported a significant decrease in systolic blood pressure in subjects with T2DM [43]. Differential responsiveness in systolic and diastolic blood pressure could not be explained by dose or study duration.

### 3.8. Overall Efficacy of Grape Seed and Bilberry and Blackcurrant Extracts to Modulate Glucose and Cholesterol Metabolism, and Blood Pressure

**Figure 7:**
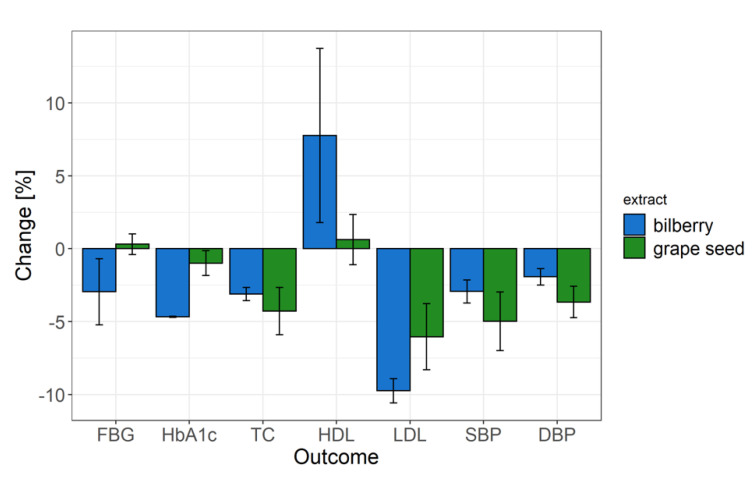
Calculated means and standard errors of percentage changes in study outcomes (FBG—fasting blood glucose, HbA1c—glycated hemoglobin, TC—total cholesterol, HDL and LDL cholesterol, SBP—systolic blood pressure, DBP—diastolic blood pressure) across all intervention studies with bilberry interventions (blue) and grape seed interventions (green).

Figure 7 shows the mean physiological changes in health outcomes upon intervention with either grape seed or bilberry and blackcurrant extract across studies as a means to evaluate their efficacy to modulate health outcomes. Intervention with bilberry and blackcurrant extract reduced HbA1c by on average 4.7% and reduced fasting blood glucose levels by on average 3%, but the variability between studies was large. Intervention with grape seed extract did not notably affect average HbA1c or fasting blood glucose levels. Both grape seed and bilberry and blackcurrant extract reduced total cholesterol by on average 4.3% and 3.1%, respectively, and LDL cholesterol levels by on average 6.0% and 9.8%, respectively. Only interventions with bilberry and blackcurrant extract increased HDL cholesterol levels by on average 7.8%, but the variability between studies was large. Both grape seed and bilberry and blackcurrant extracts reduced systolic blood pressure by 5% and 2.9%, respectively, and diastolic blood pressure by 3.7% and 1.9%, respectively, but the variability was larger, especially between grape seed extract interventions.

## 4. Discussion

We found good evidence that bilberry and blackcurrant extract lowered average levels of HbA1c in a number of longer-term Chinese studies, in subjects who were older (e.g., >50 years) and who were diagnosed with T2DM or pre-diabetes. We also found good evidence that across studies in subjects with hypercholesterolemia, T2DM or metabolic syndrome, intervention with bilberry and blackcurrant extract, and to some extent grape seed extract, for four weeks or longer, significantly improved total and LDL cholesterol levels. In addition, intervention with grape seed extract may reduce systolic and diastolic blood pressure in subjects with hypertension or metabolic syndrome. Differential responsiveness in cholesterol and blood pressure outcomes between stratified populations could not be explained by age, dose or study duration.

Thus far, a number of existing meta-analyses and a systematic review on bilberry and grape seed intervention studies have not been able to shed light on important factors that could modulate responsiveness to intervention between stratified groups of participants. One meta-analysis found that interventions with bilberry extract improved LDL and HDL cholesterol levels in subjects at risk of cardiovascular disease, in subjects with metabolic syndrome and in those with hypercholesterolemia [13], whereas a systematic review concluded that interventions with grape polyphenols did not significantly affect glycemia, insulin sensitivity, cholesterol levels or blood pressure [14]. This was in contrast to a recent meta-analysis suggesting that intervention with grape seed extract decreased LDL cholesterol and triglycerides across studies [15], and another meta-analysis suggesting that grape seed extracts decreased systolic and diastolic blood pressure, especially in subjects with metabolic syndrome and pre-hypertension [16]. Significant differences in applied inclusion and exclusion criteria between reviews in relation to study designs, study populations and outcome parameters led to inclusion of different studies, and therefore to different conclusions.

The approach taken in our critical review allowed, for the first time, the assessment of outcomes across study populations, study duration and dose, and the evaluation of responses for a wider range of outcomes including glucose and cholesterol metabolism, and blood pressure, using a correlation analysis. This enabled a better understanding of who may benefit most from grape seed and bilberry supplements, and why. Across studies, it was less clear whether factors such as intervention, dose, study population or study duration influenced the responsiveness to bilberry and blackcurrant and grape seed extracts in terms of improving total and LDL cholesterol levels, and blood pressure. The limited number of published studies with grape seed or bilberry extracts, and in some cases, questionable study and reporting quality (Figure 2), could lead to spurious conclusions. For instance, the increased likelihood of a strong correlation between glucose and HbA1c outcomes, with BMI or age, could arise from the small number of studies that reported changes in HbA1c and fasting glucose in very homogeneous study populations. Indeed, the populations assessed for changes in cholesterol outcomes and blood pressure were more heterogeneous, resulting in weak correlations between outcomes and factors relating to study design and population characteristics. Also, it is to be expected that heterogeneity in the health status of the different study populations would have affected the responsiveness to the extract interventions, with more pronounced benefits expected for those with pre-existing conditions such as T2DM and hypertension, compared with healthy populations [45,46]. For this review, we calculated average effects in inherently heterogenous populations, which could potentially lead to a high variability in outcomes [8,47]. Aggregation of raw data and evaluation of personalized data from all studies would have provided a more powerful approach to define responsiveness across stratified populations, which is of great interest when developing the next generation of treatment strategies [8].

In this review we established that longer-term interventions with bilberry and blackcurrant extract may lower HbA1c in older populations diagnosed with T2DM or pre-diabetes. In one of the two studies, bilberry and blackcurrant extract caused an average decrease in HbA1c of 5% (Figure 4) in patients with pre-diabetes and T2DM [41]. Whilst this effect was statistically significant, the efficacy of bilberry and blackcurrant extract to lower HbA1c was much lower than a reported 16% reduction in HbA1c levels after a one-year intervention with metformin in subjects with T2DM [48]. An absolute decrease of 1% in HbA1c levels above 5% has been associated with a 21% decreased risk for cardiovascular incidences, and a 25% lower mortality risk [49]. This means that an average absolute decrease of 0.3% in HbA1c levels after intervention with bilberry and blackcurrant extract [41,43] represents a 6% reduction in risk of cardiovascular incidences, and an 8% lower mortality risk. It could be argued that considering the inter-individual variability in response, the efficacy of bilberry and blackcurrant extract to lower HbA1c levels may come much closer to that of metformin in a smaller subgroup of ‘responders’. In addition, there may be scope for the use of bilberry and blackcurrant extract as an adjuvant for drug-based interventions. In this review we are not able to clarify whether the long-term efficacy of the bilberry and blackcurrant extract to lower HbA1c levels was due to the bilberry or the blackcurrant extract, or a combination of both. However, an acute study with bilberry extract increased insulin sensitivity and reduced glucose uptake in T2DM patients [50], suggesting that bilberry extract may be, at least partially, responsible for the beneficial effects on glucose metabolism. Similarly, systolic blood pressure decreased by on average 9.3 mmHg across 13 intervention studies with grape seed extract, which is equivalent to a 10% risk reduction for stroke [51]. This efficacy to reduce systolic blood pressure may make grape seed extract a potential treatment for mild hypertension.

Previous reviews and meta-analyses have compared studies without accounting for differences in intervention formulations, which emerge when working with, for example, fresh berries, berry juices, dealcoholized wine and extracts [10,13,14]. Differences in intervention formulations would be expected to affect the bioavailability of polyphenols, similar to the way in which food matrices affect bioavailability, uptake and metabolism [6]. Indeed, dietary fibers, certain minerals and proteins, carbohydrates and fats in meals can inhibit or delay polyphenol bioavailability [6,52]. For example, plasma anthocyanin glycosides peaked at later time point when strawberries were consumed with double cream, whereas plasma anthocyanin glycoside levels were decreased when a strawberry beverage was consumed with breakfast compared to before or after breakfast [6]. In addition to the food matrix, other factors such as timing of consumption, consumption with or without other foods, individual health conditions, enzyme activity and differences in gut microbiota composition are likely to affect polyphenol bioavailability, and thus exposure to the bioactive compounds [6,52]. It has to be noted that for the majority of studies included in this review, the phenolic metabolite composition of the bilberry and blackcurrant extract, or the grape seed extract, were not specified, whilst this information is essential for analyzing the relationship between the intake of supplements and its health effects.

In order to assess the role of individual factors on the responsiveness to bilberry and blackcurrant and grape seed extracts, it is paramount to understand the mechanisms and their impact. Polyphenol compounds from fruits led to an upregulation of GLUT4 glucose transporters in muscle and adipose tissue in animal and in vitro studies, thereby increasing glucose storage, phosphorylation and activation of insulin receptors, which increased insulin sensitivity and signaling, and apoptosis protection of pancreatic β-cells [53]. Furthermore, glucose transport in a Caco-2 intestinal model was decreased in the presence of glycosylated flavonoids and aglycone polyphenol compounds, suggesting reduced uptake of dietary glucose in the small intestine [54]. Consumption of anthocyanin-rich foods including berries have been associated with a reduction in cardiovascular disease risk, potentially due to mechanisms that improve flow-mediated dilation and vascular function [55]. Anthocyanins and their metabolites appear to regulate different cellular processes involved in vascular function by controlling the activity of cell signaling proteins and transcription factors and modulating gene and miRNA expression, especially in relation to modulation of inflammatory responses and platelet activation [55]. However, the majority of the mechanistic studies do not consider factors that affect transformation, absorption, digestion, metabolism and excretion of anthocyanins in the human body [6]. Indeed, a large number of in vitro studies concentrate on extracts, aglycones or parent compounds rather than anthocyanin metabolites derived from microbial metabolism, which are often present in higher concentrations in circulation, and are therefore more likely to contribute to the beneficial health effects of anthocyanin consumption [56]. Epidemiological evidence suggests that consumption of proanthocyanidins may protect, to some extent, against cardiovascular diseases and reduce free radical and peroxidative loads; however, evidence from the in vitro and in vivo studies on the involvement of proanthocyanidins in fat metabolism, obesity, and glucose pathways is currently inconsistent, mostly due to the non-standardized administration of these bioactive compounds [57].

We conclude that bilberry and blackcurrant extract may beneficially affect long-term glucose metabolism, albeit that the current evidence is only supported by a few studies in Chinese subjects with T2DM. Bilberry and blackcurrant extracts, and grape seed extracts, are likely to lower total and LDL cholesterol levels in subjects with metabolic syndrome, T2DM or hypercholesterolemia. More limited evidence suggests that grape seed extract may decrease blood pressure in hypertensive subjects or those with metabolic syndrome. Overall, we observed that based on current data, pre-existing health conditions are the main factors for determining responsiveness to intervention, and long-term interventions appear to be more beneficial for regulation of blood glucose and cholesterol metabolism. Future long-term interventions should focus on measuring responsiveness based on multiple individual factors measured in a more continuous manner, in order to better elucidate which population groups may benefit most from intervention.

## Figures and Tables

**Table 1:** Overview of supplement studies with grape seed or bilberry extract.

Intervention	Dose [mg/d]	Study Design	Study Duration [wks]	Number of Subjects	Age Range	BMI Range	Male/Female Ratio	Health Status of Subjects	Participant Drop-Out Rate (%)	Country	Reference
GSE	600	Double blind, parallel	8	40	14–28	18–25	all female	Healthy	-	Iran	[21]
GSE	400	Double blind, parallel	4	20	60+	30–36	all female	Healthy and obese	-	Iran	[22]
GSE	200	Single blind, parallel	12	53	40–62	22–27	10/11 (not considering dropouts)	Healthy, mild hypercholesterolemia	13	Japan	[23]
GSE	400	Single blind, parallel	12	53	40–62	22–27	9/11 (not considering dropouts)	Healthy, mild hypercholesterolemia	13	Japan	[23]
GSE	100	Double blind, parallel	8	96	44–56	18–24	all female	Healthy: pre-menopausal, in menopause, post-menopausal	5	Japan	[24]
GSE	200	Double blind, parallel	8	96	44–56	18–24	all female	Healthy: pre-menopausal, in menopause, post-menopausal	5	Japan	[24]
GSE	650	Double blind, cross–over with 2 week washout	4	50	44–60	23–35	25/25	Heart disease or Hypertension	-	USA	[25]
GSE	200	Double blind, parallel	8	70	39–57	–	27/43	Hypercholesterolemia	7	Iran	[26]
GSE in formulation	600	Double blind, parallel	12	180	37–57	21–31	58/34 (intervention) 45/43 (placebo)	Hypercholesterolemia	6	India	[27]
GSE	100	Double blind, parallel	8	38	-	-	-	Hypercholesterolemia	5	USA	[28]
GSE + chromium	100	Double blind, parallel	8	38	-	-	-	Hypercholesterolemia	5	USA	[28]
GSE	200	Double blind, cross–over with 8 week washout	8	42	39–57	-	18/24	Hypercholesterolemia	19	Iran	[29]
Bilberry and blackcurrant extract	320	Double blind, parallel	24	146	40–65	-	31/42	Hypercholesterolemia	3	China	[30]
GSE in formulation	540	Double blind, cross–over with 2 week washout	4	18	41–47	31–34	15/3	Metabolic syndrome	38	USA	[31]
GSE	100	Double blind, parallel	8	42	13–19	27–36	14/7 (intervention) 5/16 (placebo)	Metabolic syndrome	13	Iran	[32]
Bilberry and blackcurrant extract	320	Double blind, parallel	12	120	40–65	22–31	21/39	Metabolic syndrome	-	China	[33]
GSE	150	Double blind, parallel	4	27	42–48	34–37	4/5	Metabolic syndrome	-	USA	[34]
GSE	300	Double blind, parallel	4	27	43–51	34–39	4/5	Metabolic syndrome	-	USA	[34]
GSE	300	Double blind, parallel	12	40	31–36	27–39	4/16 (intervention) 3/17 (placebo)	Metabolic syndrome	20	Iran	[35]
GSE in formulation	1500	Double blind, parallel	52	131	58–72	23–31	all female	Postmenopausal	17	Russia	[36]
GSE	150	Double blind, parallel	16	119	44–56	24–26	17/18	Pre-hypertension	-	Italy	[37]
GSE	300	Double blind, parallel	16	119	44–56	24–26	23/14	Pre-hypertension	-	Italy	[37]
GSE	200	Double blind, parallel	12	30	40–64	19–27	2/8	Pre-hypertension	-	Japan	[38]
GSE	400	Double blind, parallel	12	30	40–64	19–27	2/8	Pre-hypertension	-	Japan	[38]
GSE	300	Double blind, parallel	8	70	62–64	18–30	19/16	Pre-hypertension	1	The Netherlands	[39]
GSE	300	Double blind, parallel	8	32	48–57	-	9/7 (intervention) 6/10 (placebo)	Pre-hypertension	-	USA	[40]
Bilberry and blackcurrant extract	320	Double blind, parallel	12	160	40–75	21–28	25/55	Pre-diabetes and T2DM	14	China	[41]
GSE	600	Double blind, cross–over with 2 week washout	4	32	55–68	24–36	16/16	T2DM	-	UK	[42]
Bilberry and blackcurrant extract	320	Double blind, parallel	24	58	56–67	20–27	17/12	T2DM	-	China	[43]
GSE	200	Double blind, parallel	8	48	30–65	25–37	-	T2DM	20	Iran	[44]

BMI—body mass index (kg/m2); GSE—grape seed extract; - missing information.

## Data Availability

All data are contained within the article.
